# XBP1-mediated transcriptional regulation of SLC5A1 in human epithelial cells in disease conditions

**DOI:** 10.1186/s13578-024-01203-x

**Published:** 2024-02-22

**Authors:** Yifei Sun, Yihan Zhang, Jifeng Zhang, Y. Eugene Chen, Jian-Ping Jin, Kezhong Zhang, Hongmei Mou, Xiubin Liang, Jie Xu

**Affiliations:** 1 Center for Advanced Models for Translational Sciences and Therapeutics, University of Michigan Medical Center, University of Michigan Medical School, Ann Arbor, MI USA; 2 The Mucosal Immunology & Biology Research Center, Massachusetts General Hospital, 55 Fruit Street, Jackson, 1402, Boston, MA 02114 USA; 3 Department of Physiology and Biophysics, University of Illinois at Chicago, Chicago, IL 60612 USA; 4 Center for Molecular Medicine and Genetics, Wayne State University School of Medicine, Detroit, MI 48201 USA

**Keywords:** ER stress, XBP1, SLC5A1, SGLT1, Epithelial cells

## Abstract

**Background:**

Sodium-Glucose cotransporter 1 and 2 (SGLT1/2) belong to the family of glucose transporters, encoded by *SLC5A1* and *SLC5A2*, respectively. SGLT2 is almost exclusively expressed in the renal proximal convoluted tubule cells. SGLT1 is expressed in the kidneys but also in other organs throughout the body. Many SGLT inhibitor drugs have been developed based on the mechanism of blocking glucose (re)absorption mediated by SGLT1/2, and several have gained major regulatory agencies’ approval for treating diabetes. Intriguingly these drugs are also effective in treating diseases beyond diabetes, for example heart failure and chronic kidney disease. We recently discovered that SGLT1 is upregulated in the airway epithelial cells derived from patients of cystic fibrosis (CF), a devastating genetic disease affecting greater than 70,000 worldwide.

**Results:**

In the present work, we show that the SGLT1 upregulation is coupled with elevated endoplasmic reticulum (ER) stress response, indicated by activation of the primary ER stress senor inositol-requiring protein 1α (IRE1α) and the ER stress-induced transcription factor X-box binding protein 1 (XBP1), in CF epithelial cells, and in epithelial cells of other stress conditions. Through biochemistry experiments, we demonstrated that the spliced form of XBP1 (XBP1s) acts as a transcription factor for *SLC5A1* by directly binding to its promoter region. Targeting this ER stress → *SLC5A1* axis by either the ER stress inhibitor Rapamycin or the SGLT1 inhibitor Sotagliflozin was effective in attenuating the ER stress response and reducing the SGLT1 level in these cellular model systems.

**Conclusions:**

The present work establishes a causal relationship between ER stress and SGLT1 upregulation and provides a mechanistic explanation why SGLT inhibitor drugs benefit diseases beyond diabetes.

**Supplementary Information:**

The online version contains supplementary material available at 10.1186/s13578-024-01203-x.

## Introduction

Sodium-Glucose cotransporter 1 and 2 (SGLT1/2) belong to the family of glucose transporters, encoded by *SLC5A1* and *SLC5A2*, respectively. SGLT2 is almost exclusively expressed in the renal proximal convoluted tubule cells, whereas SGLT1 is expressed throughout the body. SGLT inhibitor (SGLTi) drugs were originally developed based on the mechanism of blocking glucose (re)absorption mediated by SGLT1/2. Several SGLTi drugs, including Canagliflozin, Dapagliflozin, Empagliflozin, and Sotagliflozin [1–3], have gained major regulatory agencies’ approval for treating diabetes. Intriguingly, accumulating clinical data show that these SGLTi drugs provide benefits to diseases beyond diabetes, such as heart failure and renal diseases [4, 5]. In preclinical model systems, beneficial effects of SGLTi drugs were reported on a wide spectrum of diseases that include cancer, Alzheimer’s, atherosclerosis, and others [6–9]. Considering that SGLT1 is expressed globally but SGLT2’s expression is limited to the kidney, it has been speculated that the beneficial effects by SGLTi drugs in non-renal organs may be attributed to the SGLT1 inhibition.

In a previous work, we examined the SGLT1 expression level in cystic fibrosis (CF) patient-derived airway lineage cells [10]. Loss of function mutations in the cystic fibrosis transmembrane conductance regulator (CFTR) gene lead to CF, a devastating genetic disease affecting greater than 70,000 patients worldwide [11]. CFTR-F508del (dF) is the most common mutation type in CF which causes the deletion of phenylalanine at position 508 (F508) of the CFTR protein [12]. We revealed that SGLT1 is upregulated in CF bronchial epithelial (CFBE-dF) cells carrying the homozygous dF mutation [10], as well as in dF patient cell derived lung organoids [10]. These findings indicate that SGLT1 may be a therapeutic target for CF. However, the molecular basis for SGLT1 upregulation in CF conditions remains to be determined. These findings also promoted us to ask the question whether SGLT1 upregulation takes place in other human disease conditions.

Endoplasmic reticulum (ER) stress is triggered when the ER protein folding capacity is overwhelmed, often due to the excess accumulation of unfolded or misfolded proteins [13]. In the context of CF, the CFTR-F508del mutant protein misfolds [14], and is associated with elevated ER stress [15]. There is mounting evidence that ER stress response contributes to the development and progression of many diseases, including diabetes, atherosclerosis, neurodegeneration, liver diseases, and cancer [16–21]. ER stress engages the unfolded protein response (UPR), an adaptive response that reduces unfolded protein load to maintain cell viability and function [13]. The UPR is a complex signal transduction pathway that is initiated by the activation of one or more UPR transducers which include at least three: inositol-requiring protein 1α (IRE1α), protein kinase RNA-like ER kinase (PERK), and activating transcription factor 6 (ATF6). IRE1α is the most conserved UPR transducer [22]. Activated IRE1α processes the pre-matured mRNA (XBP1u) of X-box binding protein 1 (XBP1), after which the spliced transcripts (XBP1s) are translated into XBP1 protein, a transcription factor that regulates many genes involved in ER protein folding, secretion, and ER-associated degradation (ERAD) as part of a concerted effort to increase the capacity of the ER to cope with stress [23], as well as some non-canonical targets such as IL-6 in plasma cells, C/EBPα in adipocytes, and proinflammatory cytokines in macrophages [24]. XBP1 was reported to bind to cAMP-responsive elements (CRE) sites and CRE-like elements in which the core “ACGT” is highly conserved [25]. In most of its target genes, XBP1 binding occurred within 200 bp of transcriptional start sites [24]. But XBP1 targets were also enriched in additional distinct motifs including the UPR element (TGACGTG(G/A)) and the CCACG box [26, 27].

In the present work, we show that the upregulation of SGLT1 correlates with elevated ER stress markers IRE1α and XBP1 in CFBE-dF cells, and that XBP1 regulates the transcription of *SLC5A1*. We also examined other epithelial cell models under ER stress and revealed similar trend of upregulation of SGLT1. Pharmacological interference of this ER stress → SGLT1 axis by either ER stress inhibitor Rapamycin or SGLT inhibitor Sotagliflozin were effective in attenuating the ER stress response, and reduced the SGLT1 levels in these cellular models. Our work demonstrates a crosstalk between ER stress response and SGLT1 and suggests targeting this interaction for treating CF and other human diseases.

## Results

### SGLT1 and ER stress markers are upregulated in the CFBE-dF cells

**Fig. 1:**
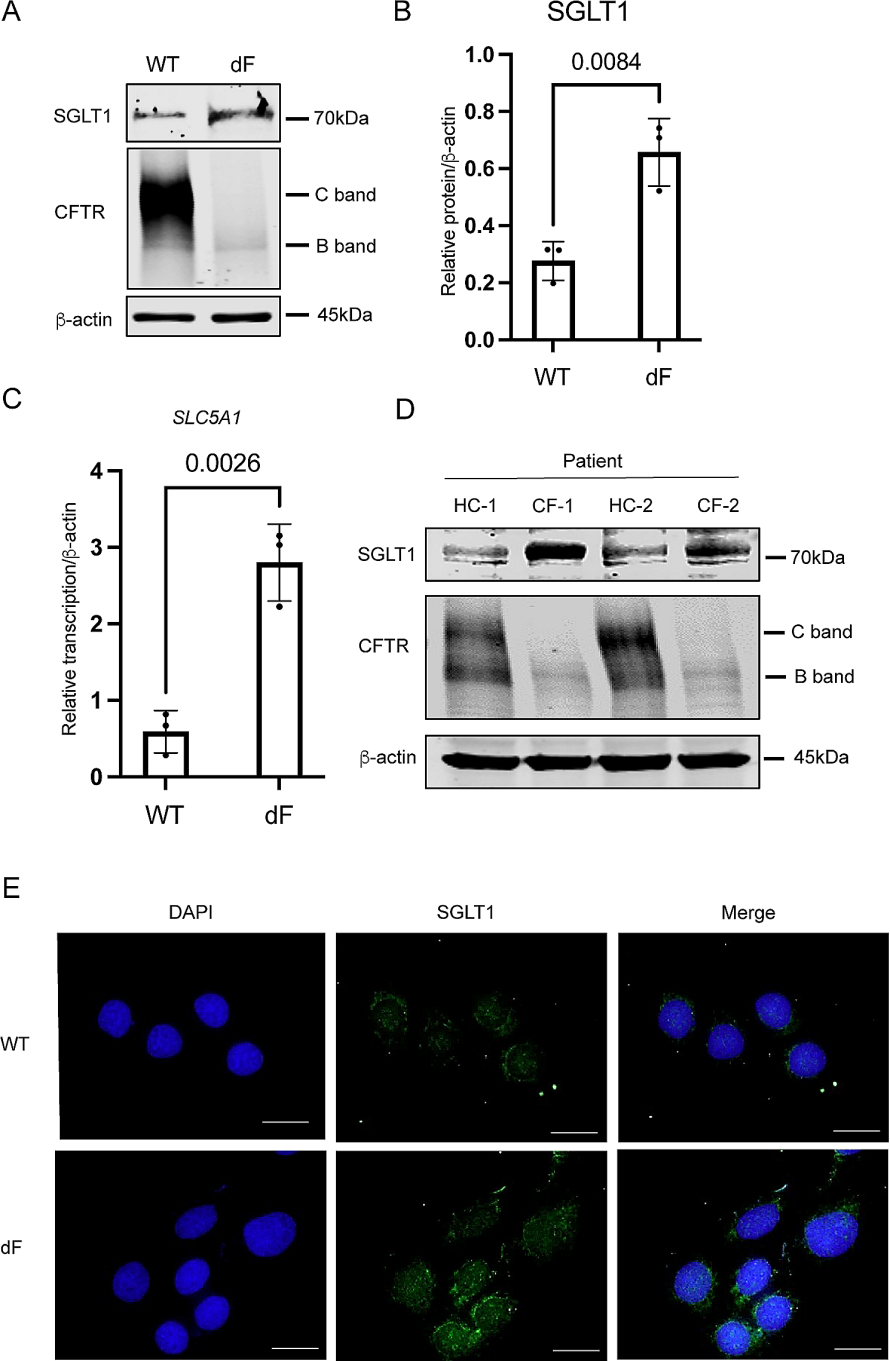
SGLT1 is upregulated in CF patient-derived airway lineage cells. (**A**) Representative gel of Western blot (WB) of SGLT1 and CFTR in the CFBE-WT cells and the CFBE-dF cells. (**B**) Quantification of WB data for the protein levels of SGLT1 in CFBE-WT cells and the CFBE-dF cells. (**C**) RT-qPCR of *SLC5A1* in the CFBE-WT cells and the CFBE-dF cells. (**D**) Western blot of SGLT1 in CF patient-derived airway epithelial cells. HC-1 and − 2: healthy control; CF-1 and − 2: CF patients of homozygous dF508 mutation. (**E**) Immunofluorescence staining of SGLT1 in the CFBE-WT cells and the CFBE-dF cells. Scale bar: 20 μm

We previously reported that SGLT1 is upregulated in different types of dF patient-derived airway lineage cells including the iPS cell-derived lung organoids, the CFBE-dF cells, and the primary airway epithelial cells [10]. In the present work, we confirmed that the levels of *SLC5A1* mRNA and SGLT1 protein were upregulated in the CFBE-dF cells compared with those in the cells of wild-type CFTR genotype (CFBE-WT) by reverse transcription-quantitative polymerase chain reaction (RT-qPCR), Western blot (WB) (Fig. 1A to C) and immunofluorescence staining (IF) (Fig. 1E). Consistently, the levels of SGLT1 protein were higher in the primary airway epithelial cells derived from two dF patients, comparing with those in two healthy control subjects (Fig. 1D).

**Fig. 2:**
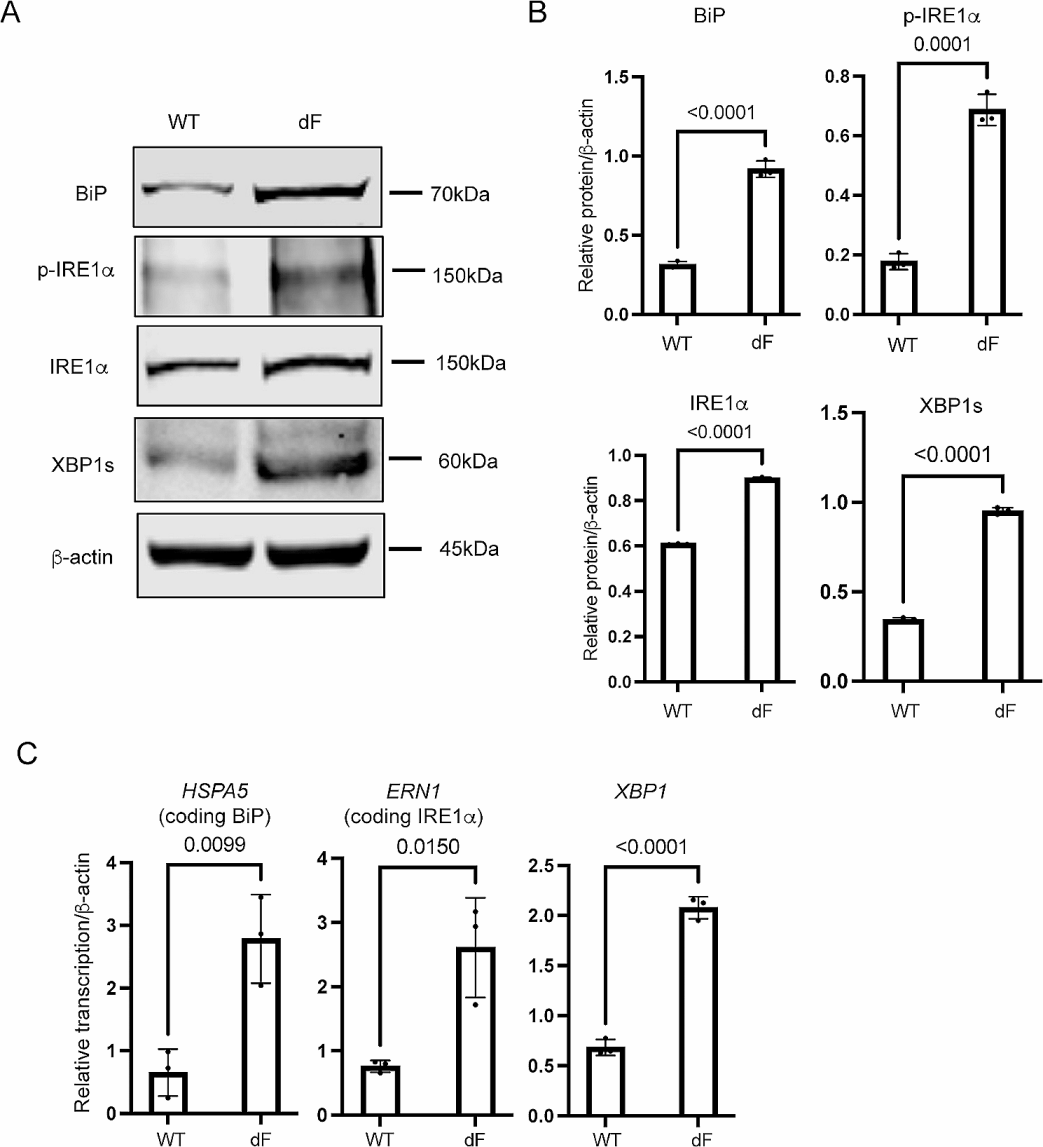
Elevation of ER stress markers in CFBE cells. (**A**) Representative Western blot (WB) gel of BiP, p-IRE1α, IRE1α, and XBP1s in the CFBE-WT cells and the CFBE-dF cells. (**B**) Quantification of WB data for the protein levels of BiP, p-IRE1α, IRE1α and XBP1s in CFBE-WT cells and the CFBE-dF cells. (**C**) RT-qPCR of *HSPA5*, *ERN1*, and *XBP1* in the CFBE-WT cells and the CFBE-dF cells

We next determined the expression levels of major ER stress markers in the IRE1α-XBP1 pathway in the CFBE cells, as previous studies have suggested that this pathway is activated in CF cells [15]. Western blot data showed that BiP, phosphorylated IRE1α (p-IRE1α), IRE1α, and XBP1s were all of higher levels in the CFBE-dF cells than those in the CFBE-WT cells (Fig. 2A and B). Consistently, the transcript levels of these ER stress markers, as well as SYVN1, DNAJB9 and ERO1A, the downstream targets of IRE1α-XBP1 UPR pathway [28, 29], were upregulated in the CFBE-dF cells compared to the CFBE-WT cells (Fig. 2C and Supplementary Fig. 1).

These data suggest a correlation between the IRE1α-XBP1 ER stress pathway markers and the SGLT1 in the CFBE-dF cells.

### XBP1 transcriptionally upregulates SLC5A1 expression in dF epithelial cells

**Fig. 3:**
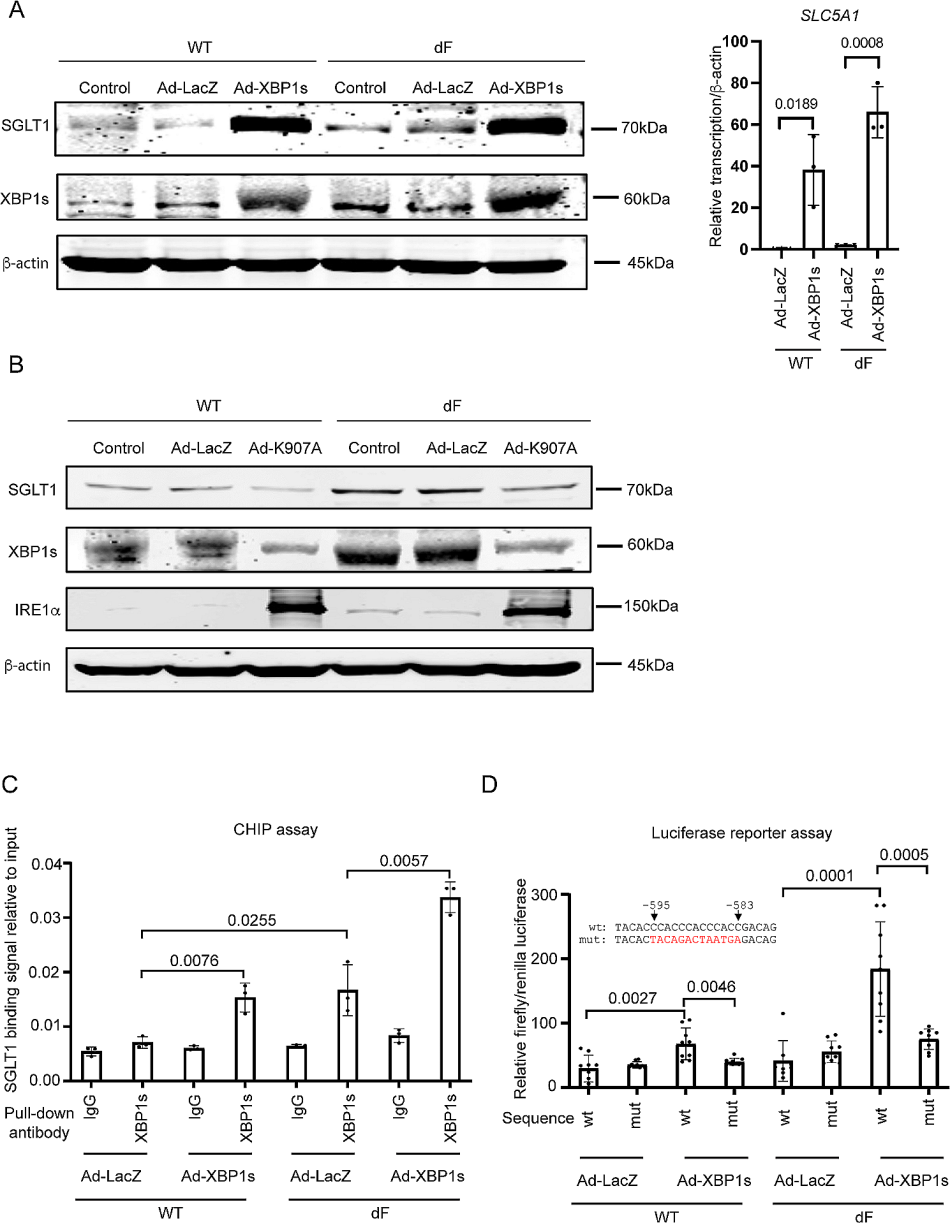
XBP1 upregulates the expression of SLC5A1. (**A**) Left: western blot of SGLT1 and XBP1s in CFBE-WT (WT) and CFBE-dF (dF) cells transduced with Ad-LacZ or Ad-XBP1s or without any viral infection (Con). Right: *SLC5A1* mRNA levels in WT and dF cells transduced with Ad-LacZ or Ad-XBP1. (**B**) Western blot of SGLT1 and XBP1s in CFBE-WT (WT) and CFBE-dF (dF) cells transduced with Ad-LacZ or AD-K907A (the dominant negative IRE1α) or without any viral infection (Con). (**C**) ChIP assay of CFBE-WT (WT) and CFBE-dF (dF) cells transduced with Ad-LacZ or Ad-XBP1s. The binding of XBP1 to the *SLC5A1* promoter was determined by qPCR. (**D**) Top: illustration of the putative wild-type (wt) binding sequence (from − 595 to -583) or the mutated binding sequence (mut) of the reporter plasmid. Red colored letters indicated mutated sequences. Bottom: relative firefly/renilla luciferase signal levels in CFBE-WT (WT) and CFBE-dF (dF) cells transfected with different combinations of the reporter plasmids (wt or mut) and the Ad viruses (Ad-LacZ or Ad-XBP1s).

We then asked the question whether XBP1 directly regulates the transcription of *SLC5A1*. In both the CFBE-WT and the CFBE-dF cells, overexpression of XBP1s by adenovirus (Ad-XBP1s) resulted in dramatically increased levels of *SLC5A1* transcripts and SGLT1 proteins, whereas minimal effects were seen when a control adenovirus overexpressing β-galactosidase (Ad-LacZ) was used (Fig. 3A).

On the other hand, overexpression of the dominant negative IRE1α carrying a K907A mutation by adenovirus (Ad-K907A) led to decreased protein levels of XBP1s and SGLT1 in both CFBE-WT and CFBE-dF cells (Fig. 3B). This is as expected based on the knowledge that IRE1α is an upstream activator of XBP1 and that IRE1α-K907A is an IRE1α dominant negative mutant that acts in a trans-dominant negative manner to inhibit endogenous IRE1α RNase activity to splice *XBP1* mRNA when it is overexpressed in cells [28, 30–32].

We then verified these findings in another cell line, the dF patient-derived pancreatic ductal epithelial (CFPAC-1-dF) cells (Supplementary Fig. 2). Like the observations in CFBE-dF cells, transduction by Ad-XBP1s dramatically elevated the protein levels of SGLT1, whereas transduction by Ad-K907A reduced the protein levels of XBP1 and SGLT1 in CFPAC-1-dF cells. Similarly when human kidney 2 (HK-2) cells, a proximal tubular cell (PTC) line derived from normal kidney, were transfected with the XBP1s overexpression vector, both the *SLC5A1* transcript and the SGLT1 protein levels were upregulated (Supplementary Fig. 3).

Together, these results suggest a IRE1α → XBP1 → SGLT1 regulatory axis in human epithelial cells, and that XBP1 directly regulate the transcription of *SLC5A1*.

### XBP1 binds to the 5’-upstream region of SLC5A1 gene promoter

We hence hypothesized that XBP1 is a transcription factor of *SLC5A1*. To test this, we worked to determine if XBP1 binds to the promoter of *SLC5A1* (Fig. 3C). Chromatin immunoprecipitation (ChIP) assay on the *SLC5A1* promoter region revealed that there are minimal XBP1 binding signals in the CFBE-WT cells (Fig. 3C). Moderate XBP1 binding signals were detected in the CFBE-dF cells, as well as in the CFBE-WT cells transduced with Ad-XBP1s (Fig. 3C). The highest XBP1 binding signals were detected, as expected, in the CFBE-dF cells transduced with Ad-XBP1s. These results show that XBP1 binds to the promoter of *SLC5A1* in CFBE cells, and such binding is activated in CF and ER stress conditions.

A previous study has suggested that XBP1 binds to the “CCACC” motif in human cells [33]. We identified a sequence “5’-CCACCCACCCACC-3’” at the − 595 to -583 bp position of the *SLC5A1* gene that contains the “CCACC” motif. To test if XBP1 binds to this sequence to exert its transcriptional regulation of *SLC5A1*, we constructed a reporter plasmid consisting of the *SLC5A1* promoter sequence (from position − 1087 to position − 21) with either the wild-type putative sequence (wt-luc) or a mutant sequence “5’-TACAGACTAATGA-3’” (mut-Luc) followed by a luciferase coding sequence. We transfected the CFBE-WT and the CFBE-dF cells with the wt-Luc or the mut-Luc plasmids, followed by transduction of these cells with Ad-XBP1s or the vehicle control Ad-LacZ. The relative luciferase signals from each of the combinations between the cell types (dF and WT) and the adenoviruses (Ad-XBP1s and Ad-LacZ) were quantified, and used as a proxy for the level of transcription induced by the XBP1’s binding to the *SLC5A1* promoter.

As expected, Ad-LacZ had no effects on the luciferase signal levels in all groups, regardless of the CFTR genotype of the cells (WT or dF) or the binding motif sequence type (wt-luc or mut-luc) of the plasmids. On the other hand, transduction of Ad-XBP1s increased the luciferase signal levels in both the CFBE-WT and the CFBE-dF cells that were transfected with wt-Luc. Such effects were abolished, however, when the cells were transfected with mut-Luc (Fig. 3D).

These data suggest that the “5’-CCACCCACCCACC-3’” sequence at the − 595 to -583 bp position of the *SLC5A1* promoter is essential for XBP1’s binding and the subsequent activation of transcription.

### Effects of targeting the ER stress → SGLT1 axis in CFBE-dF cells

We next tested whether Rapamycin or Sotagliflozin, two pharmacological inhibitors of the ER stress → SGLT1 axis, has any effects on the CFBE-dF cells. Rapamycin is an inhibitor of the mammalian target of Rapamycin (mTOR) complex 1 (mTORC1) that is known to induce autophagy; autophagy is a process that can help alleviate ER stress by removing misfolded proteins and damaged organelles [34]. As such, Rapamycin has been used as an ER stress suppressor in many studies [35–41]. Furthermore it is known that Rapamycin selectively suppresses the IRE1 signaling without affecting the other two, i.e., PERK and ATF6, pathways in human cells [39]. On the other hand, Sotagliflozin is an SGLT inhibitor. Several SGLT inhibitor drugs, including Empagliflozin, Dapagliflozin and Sotagliflozin, have gained major agencies’ approval for clinical applications [42–44]. Among them, Sotagliflozin is the most potent SGLT1 inhibitor (Supplementary Table 1).

**Fig. 4:**
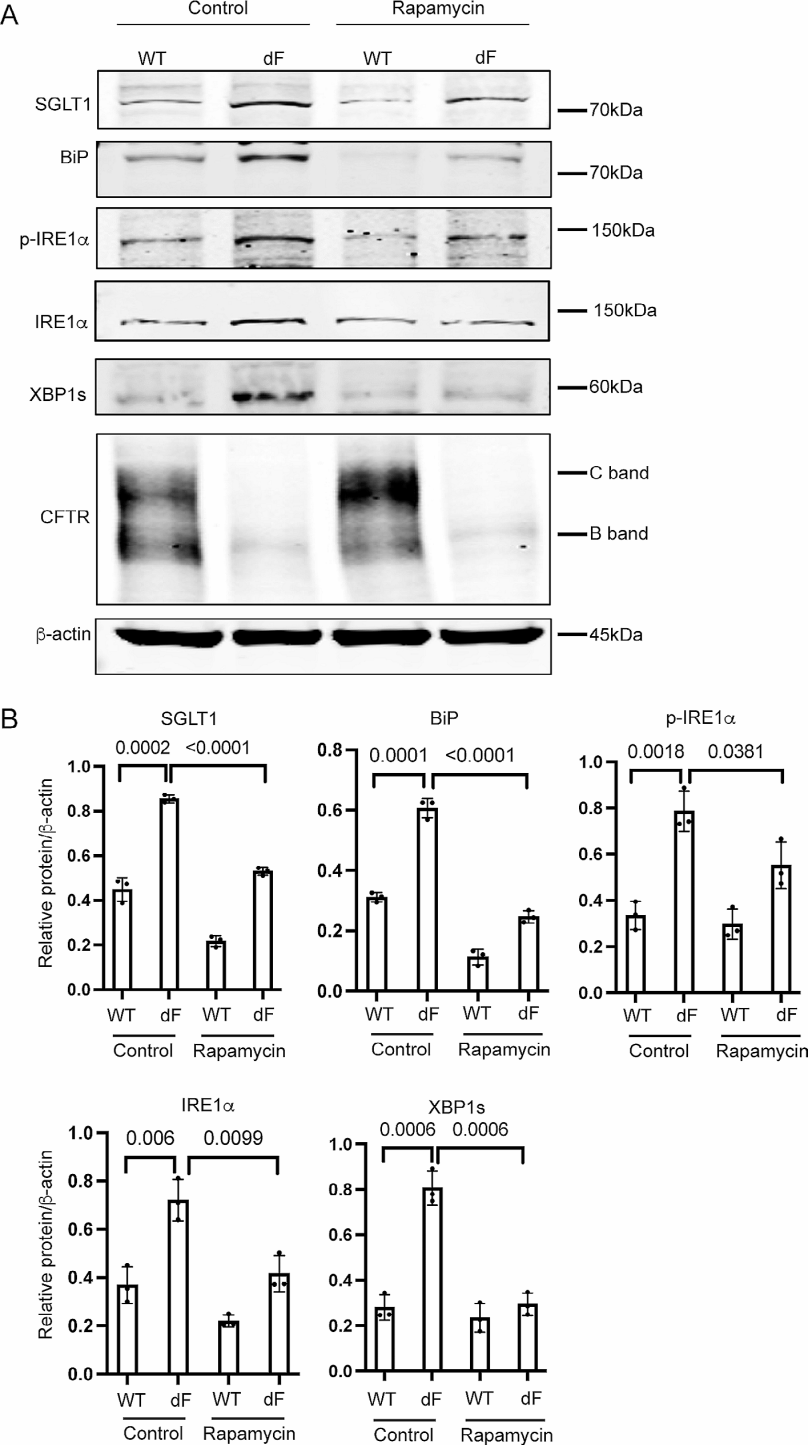
Rapamycin attenuates SGLT1 and ER stress in CFBE cells. (**A**) Representative Western blots of SGLT1, BiP, p-IRE1α, IRE1α, XBP1s and CFTR in the CFBE-WT cells and the CFBE-dF cells treated with or without Rapamycin. (**B**) Quantification of Western blots of SGLT1, BiP, p-IRE1α, IRE1α and XBP1s in the CFBE-WT cells and the CFBE-dF cells treated with or without Rapamycin

**Fig. 5:**
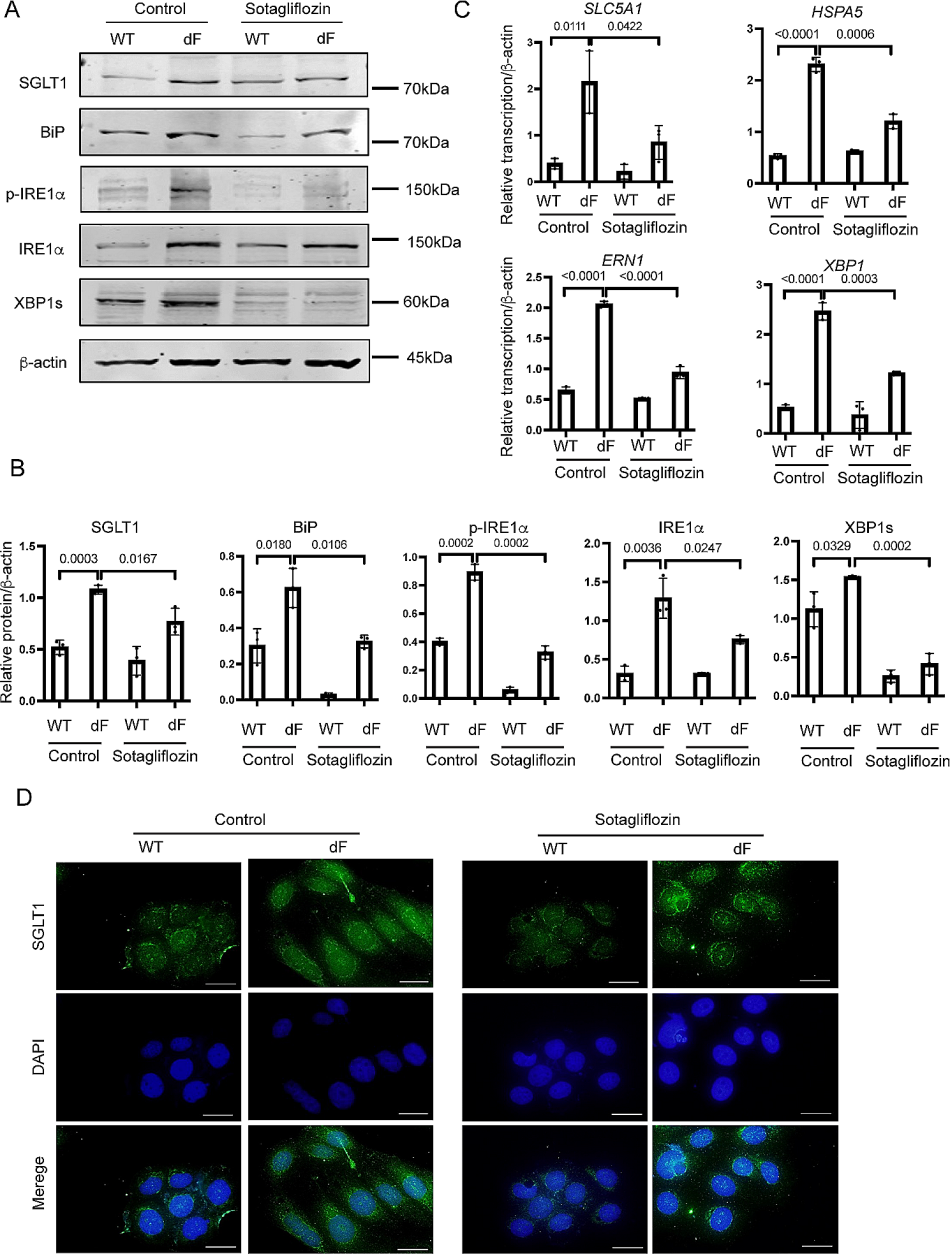
Sotagliflozin attenuates ER stress in CFBE cells. (**A**) Representative Western blot gel of SGLT1, BiP, p-IRE1α, IRE1α, and XBP1s in the CFBE-WT cells and the CFBE-dF cells treated with or without Sotagliflozin (Sota). (**B**) Quantification of WB data of SGLT1, BiP, p-IRE1α, IRE1α and XBP1s in the CFBE-WT cells and the CFBE-dF cells treated with or without Sotagliflozin. (**C**) RT-PCR of *SLC5A1*, *HSPA5*, *ERN1*, and *XBP1* in the CFBE-WT cells and the CFBE-dF cells treated with or without Sota. (**D**) Immunofluorescence staining of SGLT1 in the CFBE-WT cells and the CFBE-dF cells treated with or without Sota. Scale bar: 20 μm

Applying Rapamycin (20 nM) to the culture medium of CFBE-dF cells indeed reduced the protein levels of BiP, p-IRE1α, IRE1α and XBP1s, and consequently SGLT1 (Fig. 4A and B). Similarly, when Sotagliflozin (20 μm) was used to treat CFBE-dF cells, Western blot and RT-qPCR data showed that there were attenuated ER stress markers BiP, IRE1α, and XBP1s at both the protein and the transcription levels (Fig. 5A to C). The levels of SGLT1 protein and *SLC5A1* mRNAs were also reduced. Immunofluorescence staining confirmed this finding (Fig. 5C).

We also looked at the expression levels of key markers in the other two ER stress pathways, i.e., PERK-ARF4 and ARF6. Interestingly, although both PERK and ATF6 were upregulated in the CFBE-dF cells comparing to the CFBE-WT cells, the Sotagliflozin or Rapamycin treatments had no effects on the levels of PERK and ATF6 in these cells (Supplementary Fig. 4).

These data demonstrate potential beneficial effects of Rapamycin and Sotagliflozin on CFBE-dF cells through inhibiting the CF → ER stress → XBP1 → *SLC5A1*/SGLT1 axis.

### Effects of targeting the ER stress → SGLT1 axis in squamous epithelial cells generated in the air-liquid interface culture

ER stress is a common etiology in many human diseases. Intriguingly, SGLTi drugs appear to be effective on different human diseases. Hence, we speculated that the Disease → ER stress → XBP1 → *SLC5A1*/SGLT1 axis is not limited to CF; rather, it might exist in different human diseases as long as they are associated with ER stress in epithelial cells.

Squamous metaplasia (SQM) refers to a pre-neoplastic change of the bronchial epithelium in the lungs in response to toxic injury such as that induced by cigarette smoke [45]. During SQM, quiescent basal stem cells within the pseudostratified epithelium re-enter the cell cycle, become hyperproliferative, and begin to express markers of a squamous phenotype rather than those of the normal pseudostratified epithelium. SQM is associated with chronic obstructive pulmonary disease (COPD), and it has been reported that SQM amplifies the pathologic epithelial-mesenchymal interactions in COPD patients [46].

In a recent work, we established an air-liquid interface (ALI) culture system using patient derived airway basal cells, and demonstrated that the WNT signaling agonists CHIR-99021, when supplemented in the ALI culture (CHIR-ALI), suppressed the generation of AcTub + ciliated cells and MUC5AC + goblet cells, but drastically induced KRT13 + squamous epithelial cells, thus recapitulating key molecular and cellular changes of the SQM process in COPD [47].

**Fig. 6:**
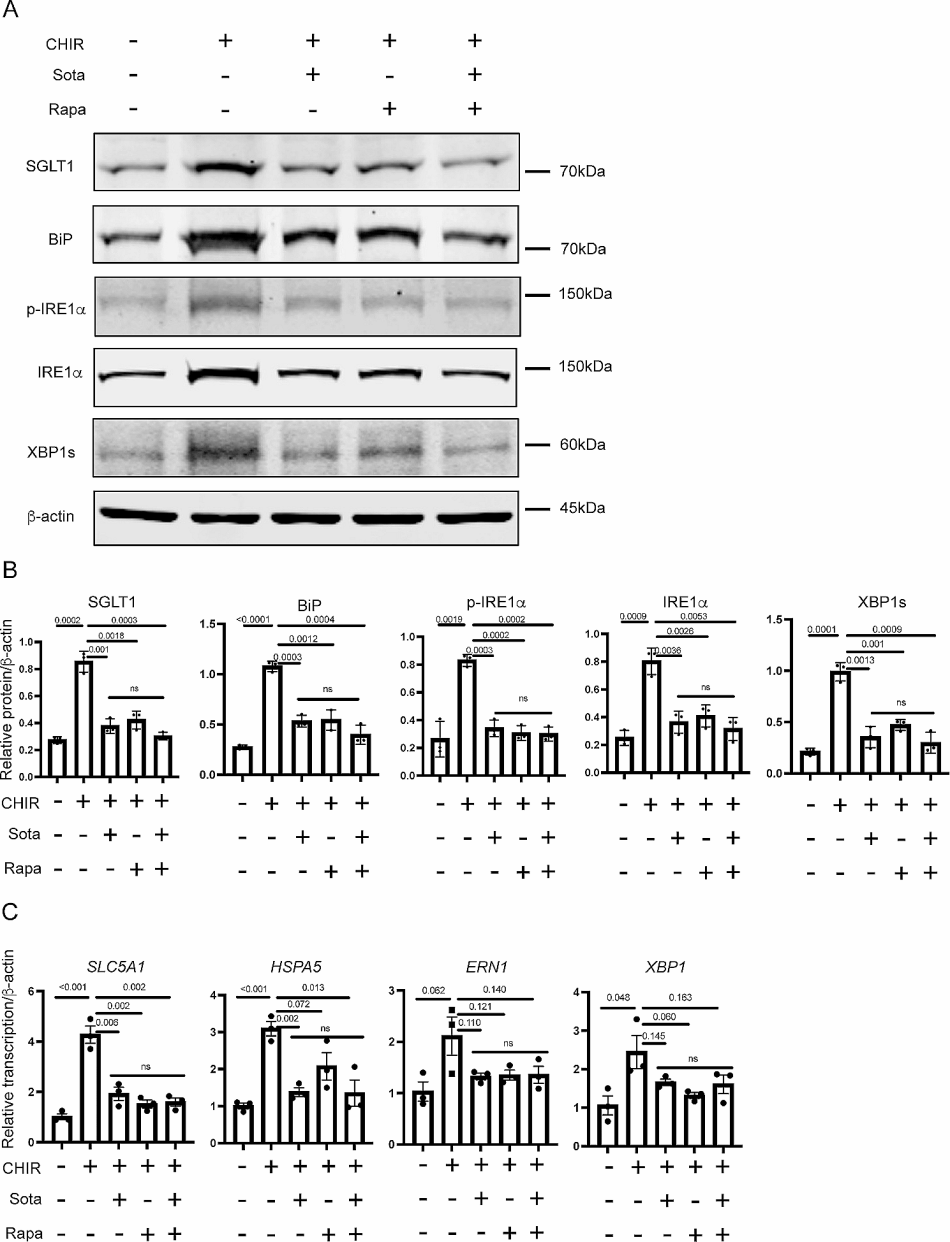
Effects of Sotagliflozin and Rapamycin on the CHIR-ALI culture. (**A**) Representative Western blot (WB) gel of SGLT1, BiP, p-IRE1α, IRE1α and XBP1s in the CHIR-ALI cells treated with different combinations of Sota and Rapamycin (Rapa). (**B**) Quantification of WB data of SGLT1, BiP, IRE1α and XBP1s in the CHIR-ALI cells treated with different combinations of Sota and Rapamycin (Rapa). (**C**) qRT-PCR of *SLC5A1*, *HSPA5*, *ERN1* and *XBP1* in the CHIR-ALI cells treated with different combinations of Sota and Rapamycin (Rapa). Control (the leftmost bar in each panel) are ALI-no-CHIR cells without Sota or Rapa supplementation to the culture medium. ns: not statistically different among these three groups

Comparison between the CHIR-ALI culture with the ALI culture without the supplementation of CHIR-99021 (ALI-no-CHIR), both derived from the airway basal cells of the same patient, revealed higher protein levels of SGLT1, and ER stress markers BiP, p-IRE1α, IRE1α, and XBP1s, as well as their corresponding mRNA levels (Fig. 6A to C) in the CHIR-ALI cells, similar to the patterns that we have observed in CFBE-dF cells.

We next examined the effects of Rapamycin and Sotagliflozin on the CHIR-ALI culture. Applying Rapamycin (10 nM) or Sotagliflozin (20 µM) to the CHIR-ALI culture both led to reduced protein and transcription levels of BiP, p-IRE1α, IRE1α and XBP1s, as well as SGLT1/*SLC5A1* (Fig. 6A to C), again a similar trend to what we have observed in CFBE-dF cells. No additive effects were observed when Rapamycin (10 nM) and Sotagliflozin (20 µM) were added together.

These results confirmed that the ER stress → SGLT1 axis exists in the CHIR-99021 induced squamous epithelial cell model and demonstrate potential beneficial effects of Rapamycin and Sotagliflozin to suppress ER stress in squamous remodeling.

### Effects of targeting the ER stress → SGLT1 axis in palmitic acid treated hepatocytes

Non-alcoholic fatty liver disease (NAFLD) refers to a spectrum of diseases ranging from steatosis to non-alcoholic steatohepatitis (NASH). ER stress response in the hepatocytes, the epithelial cells of the liver, plays a crucial role in both the onset of steatosis and progression to NASH [48, 49]. In particular, certain saturated fatty acids, such as palmitic acid (PA), can induce ER stress in human hepatocyte cell lines such as Huh-7 cells [50, 51].

**Fig. 7:**
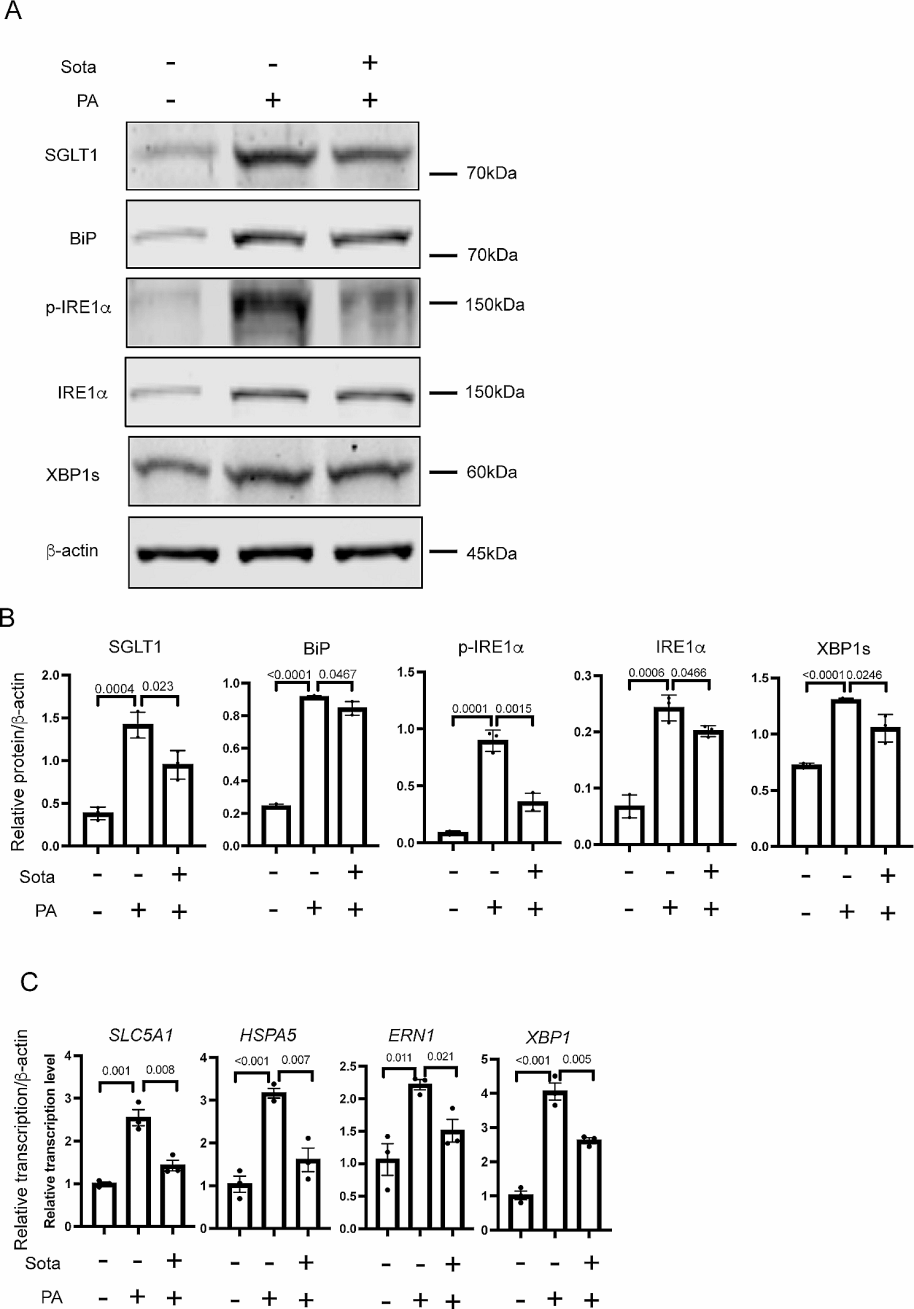
(**A**) Representative Western blot gel of SGLT1, BiP, p-IRE1α, IRE1α and XBP1s in the PA-Huh7 cells treated or without Sota. (**B**) Quantification of WB data of SGLT1, BiP, IRE1α and XBP1s in the PA-Huh7 cells treated or without Sota. Huh-7 cells were treated with BSA-complexed PA (10 µg/mL) in the presence or absence of Sota (50 µg/mL) for 36 h. Control are Huh7 cells treated with BSA vehicle without any PA or Sota treatment. (**C**) qRT-PCR of *SLC5A1*, *HSPA5*, *ERN1* and *XBP1* in the PA-Huh7 cells treated or without Sota

We first evaluated if PA treatment (10 µg/mL) to the Huh-7 cells would upregulate *SLC5A1*/SGLT1 levels, along with the anticipated activation of ER stress markers. Indeed, higher protein levels of SGLT1, BiP, p-IRE1α, IRE1α and XBP1s, and the corresponding mRNA levels were observed in the PA treated Huh-7 (PA-Huh7) cells in comparison to vehicle control treated (veh-Huh7) cells (Fig. 7A to C). Importantly, when Sotagliflozin (20 µM) were added to the PA-Huh7 cells, reduced protein and mRNA levels of BiP, p-IRE1α, IRE1α and XBP1s, as well as SGLT1/*SLC5A1* were observed (Fig. 7A to C), similar to what we have observed in the CFBE-dF and CHIR-ALI cells.

These results demonstrated the existence of hepatic ER stress → SGLT1 regulatory axis in the PA-Huh7 model and the potential beneficial effect of Sotagliflozin on attenuating PA-induced hepatic ER stress response.

## Discussion

ER stress contributes to the development and progression of many diseases, including diabetes, atherosclerosis, neurodegeneration, liver diseases, and cancer [16–21]. In the context of CF, mitigating ER stress and potentially its related inflammatory responses represents a major research direction in the drug development for this devastating disease [52, 53]. In the present work, we confirmed the existence of ER stress in CF patient-derived cells, both the airway lineage CFBE-dF cells and the pancreatic ductal epithelial CFPAC-1-dF cells. There is a prominent activation of the IRE1α-XBP1 pathway in these cells. These data corroborate with our findings in CF rabbit models, in which the IRE1α-XBP1 pathway markers are activated in all major CF affected organs including the lungs, pancreas, intestine and liver [54].

A major contribution of the current work is the establishment of a causal relationship between ER stress and SGLT1 upregulation in and beyond CF. We previously reported that SGLT1 is activated in the CFBE-dF, the CF patient iPSC-derived lung organoids, and the CF patient primary airway epithelial cells [10]. However, it is not known how SGLT1 is activated in CF conditions. Here we provided evidence that XBP1 is a transcription factor of *SLC5A1*. Furthermore, based on the data shown in Fig. 5 where SGLTi drug Sotagliflozin led to reduced ER stress markers, we expand our hypothesized regulatory axis to a regulatory loop: ER stress → XBP1 → *SLC5A1*/SGLT1 → ER stress, and speculate that disrupting a node in this loop (i.e., the SGLT1 or the ER stress) breaks this vicious cycle, which may yield benefits to CF cells and ultimately CF individuals. This idea is supported by the findings that Rapamycin and Sotagliflozin are both effective in attenuating ER stress as well as SGLT1 in the CFBE-dF cells.

Our data in CHIR-ALI and PA-Huh7 cells suggest that such regulatory loop formed by ER stress members and SGLT1 may be present in other ER stress associated diseases. Accumulated clinical data demonstrate that SGLTi drugs such as Empagliflozin, Dapagliflozin and Sotagliflozin provided benefits to a wide range of diseases, including diabetes, heart failure, and acute kidney failure [55–57]. Emerging data suggest the benefits may be broader, including inflammatory liver diseases, dementia, and stroke [58–61]. How SGLTi drugs provide therapeutic benefits to such a wide spectrum of diseases is exciting and enigmatic. The present work provides one possible mechanistic explanation, i.e., SGLT inhibition attenuates ER stress, a common etiology for many diseases [62–64], thus provides support to pharmacologically inhibiting SGLT and/or ER stress responses in these conditions.

We need to point out some limitations of the present work and future research directions. First, while we attribute the observed effects of Sotagliflozin to its SGLT1 inhibiting capacity, we cannot exclude the possibility that its SGLT2 inhibiting capacity (Supplementary Table 1) may also have contributed. Follow-up work is warranted to delineate the roles of SGLT1 and SGLT2 in attenuating ER stress in these cellular models, for example through generating *SLC5A1* and *SLC5A2* knockout CFBE-dF cells and subject these cells to different gliflozin drugs. Second, here we focused on the IRE1α-XBP1 pathway and demonstrated that it is this but not the other two ER stress pathways (e.g., PERK and ATF6) that interacts with *SLC5A1*/SGLT1. Future work should also investigate if/how modulating the PERK or the ATF6 ER stress pathways have any effects on CF. Third, although we provided mechanistic support on how diseases such as CF would lead to SGLT1 upregulation, it remains to be determined how SGLTi drug such as Sotagliflozin could in turn suppress ER stress responses as demonstrated in the present work.

There are also some technical points worth noting. For example, in the viral transduction experiments, we used Ad-K907A to overexpress the dominant negative IRE1α to suppress XBP1s. It may be helpful to include a group of Ad-WT IRE1α to overexpress the WT IRE1α, data of which will be complementary to those obtained in the Ad-K907A group to collaboratively determine the effects of the IRE1α-XBP1s axis on *SLC5A1*/SGLT1. Another interesting observation is that as shown in Fig. 3, the levels of *SLC5A1* transcripts and SGLT1 proteins elevated more in the CFBE-dF cells than in the CFBE-WT cells after viral infection with Ad-XBP1s. This may be due to the difference of baseline levels of XBP1 and SGLT1 between WT and dF cells prior to the viral transduction. It is also possible that dF cells are more responsive to stimuli from this ER stress signaling pathway. We also want to caution that the method of using CHIR-99021 to induce ER stress responses is likely only applicable to the ALI system because it is related to the squamous metaplasia process that is unique in the ALI system. It should also be noted that how CHIR-99021 induces the ER stress responses in the ALI system at the molecular level needs to be investigated in the future.

In summary, the present work reports that XBP1 is a transcription factor for SLC5A1. Our data provide a mechanistic explanation for the SGLT1 upregulation in CF and in other human disease cellular models, and provide support to target the ER stress→XBP1→*SLC5A1*/SGLT1 axis in these diseases.

## Materials and methods

### Cell culture and treatment

The CFBE-WT and the CFBE-dF cells were gifts from Dr. Fei Sun’s laboratory at Wayne State University [65]. The CFBE-WT cells were cultured in MEM media (#11095-80, Gibco, Waltham, MA, USA) supplemented with 10% fetal bovine serum (FBS) (#10,438,026, Gibco, Waltham, MA, USA) and 0.5 µg/ml puromycin (#P9620, Sigma-Aldrich, St. Louis, MO, USA) at 37 °C with 5% CO_2_ in a humidified incubator. The CFBE-dF cells were cultured in MEM media with 10% FBS and 1 µg/ml puromycin. The CFPAC-1 (#CRL-1918) cells were obtained from American Type Culture Collection (ATCC, Manassas, VA, USA), and cultured in IMDM media (#12,440,053, Gibco, Waltham, MA, USA) with 10% FBS and 100U/ml penicillin/streptomycin (#15,140,122, Gibco, Waltham, MA, USA).

Human kidney 2 (HK-2) cells were obtained from ATCC (#CRL-2190), and were cultured in DMEM-F12 media (#11,320,033, Gibco, Waltham, MA, USA) with 10% FBS.

The hepatocarcinoma cell line Huh-7 was originally provided by Dr. Charles Rice at Rockefeller University [66] and cultured in DMEM/High Glucose media containing 10% FBS.

To evaluate the effects of Sotagliflozin and Rapamycin, the CFBE-WT cells and the CFBE-dF cells were treated with 20µM Sotagliflozin (#17,011,711, Sun-Shine Chemical Technology Co., Ltd, Shanghai, China.) in serum-free MEM media for 24 h, or were treated with 20nM Rapamycin (#S1039, SelleckChem, Houston, TX, USA) in serum-free MEM media for 48 h.

To assess the effects of Sotagliflozin on attenuating hepatic ER stress response and elevation of SGLT1, Huh-7 cells were treated with 10 µg/mL fatty acid-free BSA-complexed palmitate acids (PA, #P0500, Sigma-Aldrich, St. Louis, USA) in the presence or absence of Sotagliflozin (50 µg/mL) for 36 h.

### Airway epithelial cells from CF patients and healthy control individuals

Human airway basal cells from healthy control (HC) and CF patients carrying the CFTR-F508del homozygous mutation were isolated from freshly discarded lung tissues at Massachusetts General Hospital under Institutional Review Board (IRB) protocol approval (#2017P001479 and #2013P002332). The airway basal cells were used to generate matured airway epithelial cells in the air-liquid interface (ALI) culture, as previously described [67]. The matured airway epithelial cells from HC and CF individuals were used for the analysis of gene and protein expressions. to promote squamous remodeling, 1 µM CHIR99021 (#4423, Tocris Bioscience, Minneapolis, MN) was added to the ALI medium as previously described [47]. After 14 days of differentiation, epithelial cells were treated with vehicle, 20 µM Sotagliflozin and 10 nM Rapamycin, individually or in combination, for 3 days before cell harvest for protein and RNA extraction.

### Antibodies

Antibodies against Bip/GRP78 (#3177), IRE1α (#3294), and β-actin (#3700) were obtained from Cell Signaling Technologies (Danvers, MA, USA). The CFTR antibody (#217 and #596) was from the Cystic Fibrosis Foundation Therapeutics (Bethesda, MD). The SGLT1 antibodies were from Invitrogen (PA5-88282, Waltham, MA, USA; for Western blot) and Abcam (ab14686, Waltham, MA, US; for immunofluorescence staining). The p-IRE1α antibody (#AP0878) was from ABclonal Technology (Woburn, MA, USA). The XBP1s antibody (#619,502) was from BioLegend (San Diego, CA, USA). The secondary antibodies were from LI-COR Biosciences (#D01216-10 and #D00226-05, Lincoln, NE, USA; for Western blot) and Jackson ImmunoResearch Laboratories (#147,158, West Grove, PA, USA; for immunofluorescence staining).

### Protein extraction and Western blot

Cells were lysed in RIPA lysis buffer (#89,900, ThermoFisher Scientific, Waltham, MA, USA) supplemented with protease inhibitor and phosphatase inhibitor cocktails (#11,873,580,001, Roche, Penzberg, Germany). Proteins were resolved in 10% SDS–PAGE gels and transferred to nitrocellulose membranes (Bio-Rad, Hercules, CA, USA). Membranes were blocked in TBST containing 5% non-fat milk at room temperature for 2 h, and were incubated with primary antibodies (1:1000 dilution) at 4 °C overnight. After washing with TBST, membranes were incubated with secondary antibodies (1:8000 dilution) at room temperature for 1 h. After TBST wash, bands were scanned and quantified using an Odyssey Infrared Imaging System (LI-COR Biosciences, Lincoln, NE, USA).

### RNA isolation and quantitative real-time PCR (qRT-PCR)

Total RNA from cell samples was extracted using the Trizol reagent (#15,596,018, ThermoFisher Scientific) and purified with the RNeasy kit (#74,106, QIAGEN, Hilden, Germany). RNAs were reverse transcribed into cDNA with the SuperScript III kit (#18,080,051, ThermoFisher Scientific). The target gene expression at the transcription level was assessed by the quantitative real-time PCR system (Bio-Rad, Hercules, CA, USA) using iQ SYBR Green Supermix (#1,708,884, Bio-Rad, Hercules, CA, USA). β-actin was used as the internal control. Primers for qRT-PCR are listed in Supplemental Table 2.

### Immunofluorescence (IF) staining

The CFBE-WT cells and the CFBE-dF cells were fixed on Nunc Lab-Tek Chamber Slides (Thermo Fisher Scientific) with 4% paraformaldehyde in PBS for 15 min. After washing with PBS, the slides were blocked with 5% donkey serum for 1 h at room temperature and then incubated with primary antibody against SGLT1 (1:50 dilution). After PBS washing, the slides were incubated with Alexa Fluor–labeled secondary antibody (1:1000 dilution) at room temperature for 1 h. IF slides were washed with PBS and mounted before image collection. IF images were acquired using an Olympus IX73 microscope.

### Adenoviral infection

Adenovirus expressing spliced XBP1 (Ad-XBP1s) and adenovirus expressing mutant IRE1 K907A (Ad-K907A) were produced as previously described [32]. For XBP1s overexpression, the CFBE-WT cells and the CFBE-dF cells at approximately 70–80% confluency were infected with Ad-XBP1s at a MOI of 100 for 48 h. To suppress XBP1 expression, the cells were transduced with Ad-K907A at an MOI of 100 for 48 h. The adenovirus encoding LacZ (Ad-LacZ) infection or non-infection served as controls.

### Chromatin immunoprecipitation (ChIP) assay

The CFBE-WT cells and the CFBE-dF cells were transduced with Ad-XBP1s at an MOI of 100 for 48 h to overexpress XBP1s. Ad-LacZ was used as the vehicle control. ChIP assay was performed with the SimpleChIP® Enzymatic Chromatin IP Kit (#9003, Cell Signaling Technologies, Danvers, MA, USA), following the manufacturer’s instructions. The DNA/protein complex was immunoprecipitated with control IgG or with anti-XBP1s antibody. Purified precipitated DNA was used as the template for qPCR. Primers used to detect the putative XBP1s binding motif on human SLC5A1 promoter are listed in Supplemental Table 2.

### Plasmid construction and transfection

The putative promoter region of the human *SLC5A1* gene (from position − 1087 to position − 21) was PCR-amplified from human genomic DNA by using forward primer 5’-TTTCTGTGGTCCTCTGCCTC-3’ and reverse primer 5’-TCCTTATACGGCCTCCTGGT-3’. The amplified promoter region was inserted into the pGL4.10 luciferase reporter vector (#E1910, Promega, Madison, WI, USA) by using In-Fusion® HD Cloning Kit (#102,518, Takara, CA, USA). The 5’-CCACCCACCCACC-3’ box on the human SLC5A1 promoter (-595 to -583), which is the putative XBP1s binding site, was mutated to 5’-TACAGACTAATGA-3’ by using Q5 SiteDirected Mutagenesis Kit (#E0554S, New England Biolabs, Ipswich, MA). All plasmids were validated by Sanger sequencing. The plasmids were named wt-Luc and mut-Luc, respectively.

The CFBE-WT cells and the CFBE-dF cells at 70–80% confluence were transfected with either the wt-Luc or the mut-Luc plasmids by lipofectamine 2000 (#11,668,019, ThermoFisher Scientific) according to the manufacturer’s protocol. After 24 h, these cells were infected with Ad-LacZ or Ad-XBP1s (MOI, 100). Luciferase activity was detected using Dual Luciferase Reporter Assay System (#E1910, Promega, Madison, WI, USA).

### Statistics analysis

Statistical analyses were performed using GraphPad Prism version 8.0 (GraphPad Software, San Diego, CA, USA). Data were reported as mean ± SEM (standard error of means) with three replicates for each data point. Comparison between two groups was analyzed by unpaired, 2-tailed Student’s t test (GraphPad).

### Electronic supplementary material

Below is the link to the electronic supplementary material.

**Supplementary Material 1:** Supplementary Figures and Tables

## Data Availability

All data analyzed in this study are provided in this article and its additional files. All data used in this study are available from the corresponding author on reasonable request.
