# Supplementary material for: XBP1-mediated transcriptional regulation of SLC5A1 in human epithelial cells in disease conditions
Source: Cell Biosci. 2024 Feb 22;14:27. doi: 10.1186/s13578-024-01203-x (PMC10885492; doi:10.1186/s13578-024-01203-x)
Supplement: Supplementary file 1 — Supplementary Material 1: Supplementary Figures and Tables [file 13578_2024_1203_MOESM1_ESM.docx]

**Supplementary Data**

**Title**: XBP1-mediated transcriptional regulation of SLC5A1 in cystic fibrosis bronchial epithelial cells

**Supplementary Figure 1.** Transcription levels of the targets of the IRE1α-XBP1-mediated UPR pathway, including *SYVN1*, *DNAJB9*, *ERO1A*, *ERO1B*, and *EDEM1*, in CFBE-WT and CFBE-dF cells.

**
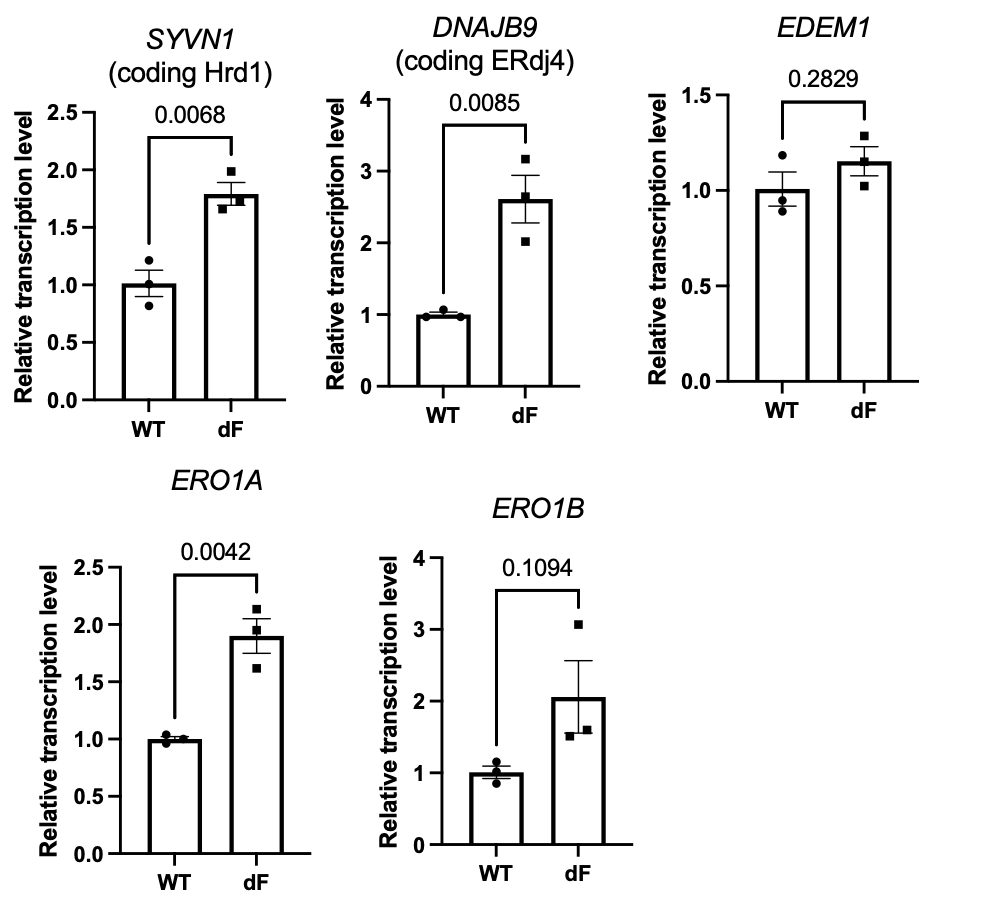
**

**Supplementary Figure 2.** XBP1 upregulates SGLT1 expression in CFPAC-1-dF cells. (A) CFPAC-1-dF cells were infected with Ad-LacZ or Ad-XBP1s for 48 hours. SGLT1 and XBP1s protein levels were determined by western blot. (B) CFPAC-1-dF cells were infected with Ad-LacZ or Ad-K907A for 48 hours. SGLT1, XBP1s, and IRE1α protein levels were determined by western blot.


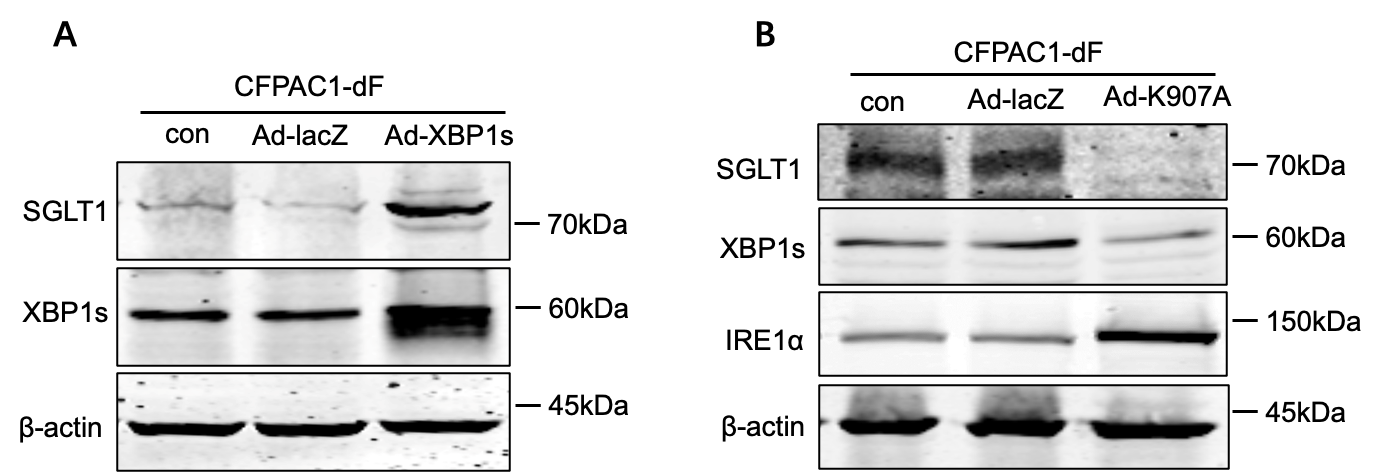


**Supplementary Figure 3.** XBP1 upregulates SGLT1 expression in HK-2 cells. (A) Western blot of XBP1s, SGLT1 and β-actin in human kidney 2 (HK-2) cells transfected with the overexpression vector pcDNA-XBP1s or the control empty vector (Vector). (B) Transcription levels of *SLC5A1* in HK-2 cells transfected overexpression vector pcDNA-XBP1s or Vector.

**
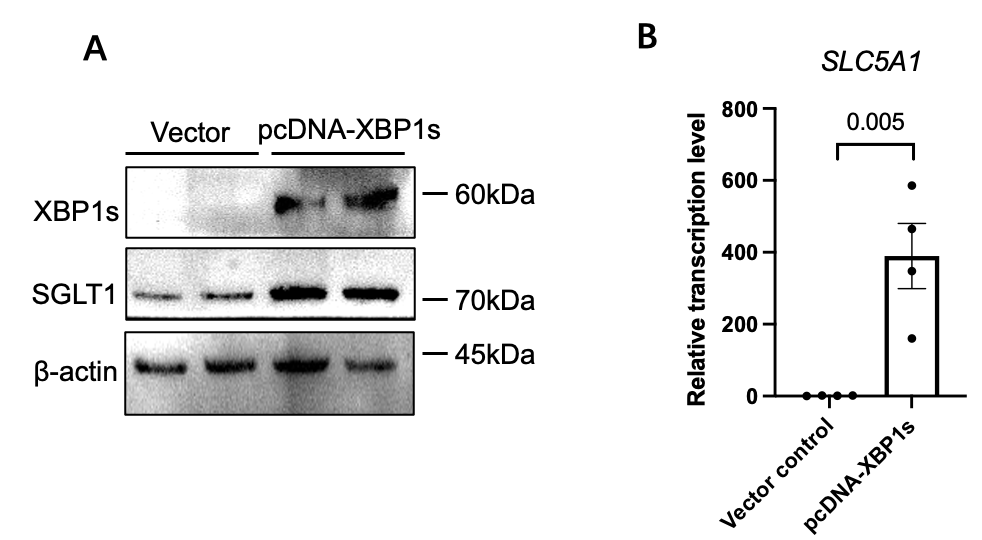
**

**Supplementary Figure 4.** Sotagliflozin and rapamycin did not affect other ER stress pathways in CFBE cells. (A) Representative western blot (WB) gel of PERK and ATF6 in CFBE-WT and CFBE-dF cells treated with vehicle control (Con) or Sotagliflozin (Sota). (B) Quantification of WB data of the PERK and ATF6 protein levels in CFBE-WT and CFBE-dF cells treated with vehicle control (Control) or Sotagliflozin (Sota). (C) Representative western blot (WB) gel of PERK and ATF6 in CFBE-WT and CFBE-dF cells treated with vehicle control (Con) or Rapamycin (Rapa). (D) Quantification of WB data of the PERK and ATF6 protein levels in CFBE-WT and CFBE-dF cells treated with vehicle control (Control) or Rapamycin (Rapa).

**
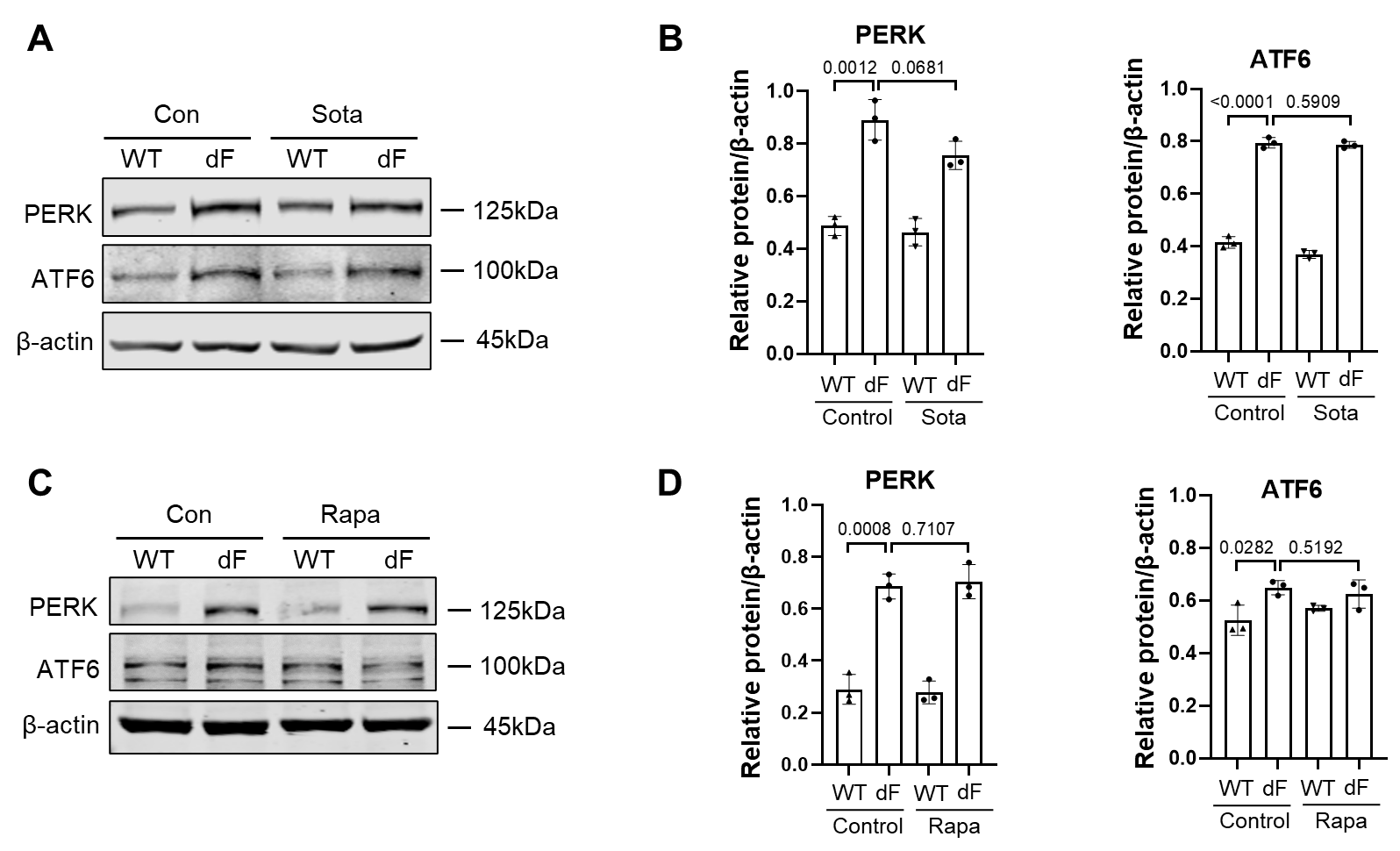
**

**Supplementary Table 1 (adapted from supplementary ref (1))**

| Drug | IC50 for SGLT2 (nM) | IC50 for SGLT1 (nM) | FDA/EMA approval year |
| --- | --- | --- | --- |
| Empagliflozin | 3.1 | 8,300 | FDA 2014; EMA 2014 |
| Ertugliflozin | 0.9 | 1,960 | FDA 2017 |
| Dapagliflozin | 1.2 | 1,400 | FDA 2014; EMA 2012 |
| Canagliflozin | 2.7 | 710 | FDA 2013; EMA 2013 |
| Sotagliflozin | 1.8 | 36 | FDA 2023; EMA 2019 |

**Supplemental Table 2: primers used in the present work**

| Gene | Primer sequences for qPCR (h=human) |
| --- | --- |
| *SLC5A1* | Forward: TCCTCACCAAACCCATTCCG |
|  | Reverse: TCCGCATCCAGGTCAATACG |
| *HSPA5* | Forward: CCTGGGTGGCGGAACCTTCGATGTG |
|  | Reverse: CTGGACGGGCTTCATAGTAGACCGG |
| *ERN1* | Forward: AGAGAAGCAGCAGACTTTGTC |
|  | Reverse: GTTTTGGTGTCGTACATGGTGA |
| *XBP1* | Forward: CCGCAGCAGGTGCAGG |
|  | Reverse: GAGTCAATACCGCCAGAATCCA |
| *GAPDH* | Forward: TGAAGGTCGGAGTCAACGG |
|  | Reverse: AGAGTTAAAAGCAGCCCTGGTG |
| *SYVN1* | Forward: CTTCACCGTTTTTCGGGATGA |
|  | Reverse: CCAGGAGGAACATAAGAGAGACA |
| *DNAJB9* | Forward: TCTTAGGTGTGCCAAAATCGG |
|  | Reverse: TGTCAGGGTGGTACTTCATGG |
| *ERO1A* | Forward: GACTGTGCTGTCAAACCATGT |
|  | Reverse: CCAAGTCGTTCAGCTTGTTCA |
| *ERO1B* | Forward: TTCTGGATGATTGCTTGTGTGAT |
|  | Reverse: GGTCGCTTCAGATTAACCTTGT |
| *EDEM1* | Forward: GCTACGACAACTACATGGCTC |
|  | Reverse: GACTTGGACGGTGGAATCTTT |
| *SLC5A1* ChIP | Forward: GTTTCTTCCTCTTTACAGTGGGGG |
|  | Reverse: CCAGCACAATCCTAATCTCTTGGC |

**References in the Supplementary Data**

1. Q. Zeng *et al.*, Mechanisms and Perspectives of Sodium-Glucose Co-transporter 2 Inhibitors in Heart Failure. *Front Cardiovasc Med* **8**, 636152 (2021).
